# Correlation Analysis Between Physical–Chemical and Biological Conditions in the River and the Incidence of Diseases in the City of Piracicaba, Brazil

**DOI:** 10.3390/toxics13050359

**Published:** 2025-04-30

**Authors:** Alexander Ossanes de Souza, Deoclecio Jardim Amorim, Ernani Pinto

**Affiliations:** Center of Nuclear Energy in Agriculture, Av. Centenário, 303—São Dimas, University of São Paulo, Piracicaba 13416-000, SP, Brazil; amorim@cena.usp.br

**Keywords:** respiratory diseases, neurological symptoms, environmental contamination, seasonal health risks, water quality, public health

## Abstract

The Piracicaba River basin, in the State of São Paulo, Brazil, covers approximately 12,400 km^2^ and plays a crucial economic role in São Paulo’s agribusiness corridor. However, it faces recurrent episodes of pollution, impacting water quality and public health, especially in urban areas exposed to contamination. Despite this, few studies have investigated the ecological and epidemiological consequences of this environmental degradation. Therefore, this study analyzed the correlation between physicochemical and biological variables of the Piracicaba River and the incidence of diseases in the city of Piracicaba between January 2019 and September 2024. Data on hospital admissions for respiratory, neurological, and liver symptoms were used, as well as environmental and water quality information, such as dissolved oxygen, turbidity, conductivity, and the presence of cyanobacteria, obtained from public databases. The results showed seasonal patterns and long-term trends, highlighting the health risks associated with the river’s pollution. Parameters such as phosphorus, pH, cyanobacteria concentration and climatic factors (temperature and humidity) showed an influence on the occurrence of respiratory, digestive, and neurological diseases. The study reinforces the need for continuous monitoring of water quality and public policies to mitigate impacts on the population’s health.

## 1. Introduction

The Piracicaba River originates in the municipality of Americana, from the confluence of the Jaguari and Atibaia rivers, and its mouth is next to the Tietê River, in the municipality of Barra Bonita. The river basin (formed by the sub-basins Atibaia, Jaguari and Piracicaba) covers an area of approximately 12,400 km^2^ [1,2]. Although it plays a crucial economic role in the region, it has experienced recurrent contamination events with ecological and public health consequences. It is continuously monitored by the Environmental Company of the State of São Paulo (CETESB) and the Municipal Water and Sewage Services (SEMAE), revealing significant declines in water quality over the years, particularly within Piracicaba [1,2]. During low-flow periods, high levels of turbidity, organic carbon, and *Escherichia coli*, together with persistent hypoxia, have been reported [1,2]. These conditions are further exacerbated by sediment runoff, urban discharge, and agricultural inputs, leading to cumulative pollutant loads [1,3,4].

Recent events highlight the severity of this degradation. In December 2023, a cyanobacterial bloom (*Microcystis* sp.) caused the river Piracicaba to exhibit a greenish coloration, coinciding with elevated microcystin levels [2,5]. This was followed by a mass fish mortality event in January 2024, attributed to hypoxic conditions and toxin release [2,5]. One of the main ways in which humans are exposed to cyanotoxins is through the ingestion of contaminated drinking water or the consumption of aquatic products. The most common cyanotoxins include microcystins, which can affect the liver, and anatoxins and saxitoxins, which affect the central nervous system, among others. Some studies indicate that aquatic organisms can accumulate toxins in their tissues, which can lead to food poisoning [6,7]. In addition, these episodes highlight the risk associated with aerosolized cyanobacteria and allergenic molecules, among other parameters known to aggravate respiratory, neurological, and hepatic conditions [8,9]. The research by Cheng et al. [10] concluded that aerosols containing microcystin in water can be transferred to the air through a bubble bursting process. The researchers report that humans can come into contact with aerosols containing microcystin when performing recreational activities in the area [10]. In the study by Labohá et al. [11], the authors detected components of cyanobacterial blooms in ambient aerosols above the surface layer of freshwater bodies. Lipopolysaccharides (LPS) could be detected in the thoracic/respirable fraction (0.1–10 µm). The study made it possible to demonstrate that LPS derived from phytoplankton or cyanobacterial blooms induced releases of pro-inflammatory cytokines from human bronchial epithelial cells in vitro; in addition, both the extracellular and intracellular fractions of the water sample from a reservoir with cyanobacterial bloom were also able to induce pro-inflammatory responses in human bronchial epithelial cells, at dilutions close to the concentrations in the original water [11]. In the review article proposed by Vejerano et al. [12], the authors highlight the importance of studying harmful algal blooms (HABs) and further report that aerosolized toxins are vulnerable to atmospheric processing, and can degrade and produce byproducts with varying potencies in comparison. Comorbid factors, such as exposure to air pollutants due to increased commercial activities in ports, may represent a significant exposure pathway for a considerable portion of the global population [12].

River pollution often leads to an increase in cases of disease in the general population, as described by Shultana and Khan [13]. There has been an increase in cases of waterborne diseases. Among these diseases, we can highlight dermatitis and respiratory diseases [13]. In Katakwar’s [14] study, it is highlighted that river pollution has been one of the major environmental issues in the riverine villages of Narmada and the urban area of Hoshangabad. The analysis of pH, color, turbidity, biochemical oxygen demand (BOD), hardness, total dissolved solids (TDS), chloride (Cl_2_), carbon dioxide (CO_2_), and chemical oxygen demand (COD) presents a variety of correlated diseases [14]. In the study by Kormoker et al. [15], 17 water quality parameters (electrical conductivity, pH, total suspended solids, temperature, F^−^, Cl^−^, SO_4_^2−^, Cr, Ni, As, Cd, Hg, Cu, Pb, Fe, Mn, and Zn) of surface and deep waters of the Buriganga River were evaluated, and it was observed that the water is unsafe for residential and recreational uses. They observed, through analysis of main components and correlation, that water quality is controlled by pollution from the combustion of lubricating oils, fuel additives, vehicle exhaust gases, domestic wastewater, and inorganic fertilizers from agricultural fields. In addition, they observed that water conditions and the concentration of some chemical elements can cause adverse effects on human health [15].

The Piracicaba River’s recurring pollution events provide an opportunity to address this gap, making a correlation between pollution events and the increase in cases in the public health system. Mainly because it is an important water source and a recreational center, it attracts residents and tourists to places such as “Rua do Porto” [1,2]. Recreational activities during contamination episodes increase exposure to toxins and other contaminants, but the health impacts of transient populations remain undocumented, potentially underestimating the true burden of pollution-related health problems [9,10,11,12]. Since the Piracicaba River is an important source of water for the region, it is extremely important to study the correlation between the quality of the river’s water and the recurring diseases in the city. For this purpose, statistics are an excellent tool, which allows us to analyze the variables of the river’s water quality and climatic factors and associate them with the incidence of some diseases reported by the Unified Health System (SUS) [16].

Our study highlights the critical need for integrated approaches to mitigating the health impacts of waterborne pollution in the Piracicaba River. The river crosses urbanized zones and interacts directly with the population that resides in close proximity to the riverbanks or engages in activities that place them in frequent contact with the river environment, such as fishing, bathing, and recreation in areas like Rua do Porto and the municipal park near the waterfall. Despite our continuous monitoring of regular water quality parameters, such as pH, dissolved oxygen (DO), turbidity, and nitrogen, among others, there is still a lack of studies that correlate these parameters with cases of hospitalizations in the SUS (Unified Health System) [1,2,17,18,19]. Seasonal spikes in hospital admissions highlight the need for responsive health systems to manage increased patient loads during pollution [16]. Awareness campaigns and restrictions on recreational activities near the river during periods of high risk can protect vulnerable populations, as recommended by WHO guidelines [15].

We hypothesize that acute exposure to air (from contaminated aerosols) and water pollution significantly increases hospital admissions for respiratory, liver, and neurological diseases, among others, while long-term exposure contributes to chronic health problems. Our study assessed acute impacts by correlating pollution events with immediate increases in hospital admissions. We also assessed long-term effects by analyzing cumulative health impacts from January 2019 to September 2024, focusing on chronic trends linked to repeated exposure to pollutants. Finally, we inform a new policy and provide recommendations for monitoring actions and public health interventions to reduce exposure risks during periods of contamination.

## 2. Materials and Methods

### 2.1. Study Design

This study was designed as a single-center, analytical ecological observational study, focusing on cases within the city of Piracicaba. Data were obtained from the largest public health database in Brazil, DATASUS, managed by the Brazilian Ministry of Health. DATASUS provides comprehensive health information through the Unified Health System’s Information Technology Department [16], making it a reliable source for public health analysis.

The study evaluated the rates of hospital admissions due to liver, digestive, and respiratory diseases, among others, analyzing variations between January 2019 to September 2024. For analysis, all conditions classified under liver, respiratory diseases, neurological diseases, or other diseases related to contact with cyanotoxins according to the International Classification of Diseases (ICD-10) were included. Respiratory diseases were chosen for this study due to the potential of aerosols to transport toxic compounds. Hospital admission data were extracted from Authorizations for Hospitalization (AIHs) containing ICD codes.

In addition to health data, information was collected from the Cantareira System, a series of reservoirs supplying water to the Piracicaba region and the Greater São Paulo area. The PCJ situation room was used to monitor the flow of the Piracicaba River [17]. Flow data, reported as cubic meters per second (m^3^/s), level flow (m), and precipitation (mm), were converted into monthly averages to align with the periods of hospital admissions data. In addition, data on temperature, wind speed, and relative humidity were obtained by the National Institute of Meteorology (INMET) and converted into monthly averages [18]. Data on pH, dissolved oxygen, turbidity, nitrogen, phosphorus, cyanobacteria, and chlorophyll were obtained from the website of the Municipal Water and Sewage Service (SEMAE) of the city of Piracicaba [2]. This synchronization facilitated a more direct comparison, focusing on short-term pollution exposure episodes.

### 2.2. Study Population and Setting

This study analyzed the population of Piracicaba, Brazil, admitted to hospitals or clinics with symptoms related to liver, respiratory, neurological, or other related diseases. The study imposed no restrictions concerning age, gender, or the type of healthcare facility (public or private) as long as the facility was registered with ANVISA. While transient populations, such as tourists, might have been included, it was not possible to differentiate or exclude these individuals from the dataset.

This retrospective, descriptive, and analytical study utilized secondary data available in DATASUS, the official database of the Brazilian Unified Health System (SUS) [16]. The population studied included all cases reported between the defined periods (2019 to 2024) in Piracicaba. Exclusion criteria were cases reported outside the city of Piracicaba. Cases with incomplete data that could not be matched or verified in the SUS system.

This study adhered to the ethical norms of Resolution 466/12 [20]. There was no need for submission to a Research Ethics Committee or the application of an Informed Consent Term, as all data used were secondary, publicly available, and anonymized, with no identifiable information regarding individuals.

### 2.3. Data Sources and Collection

Health and environmental data for this study were sourced from publicly available databases and monitoring systems, ensuring alignment with the study objectives and defined timeframes. The following data sources and processes were utilized:

#### 2.3.1. Health Data

Hospitalization data were obtained from the DATASUS database, specifically from the “Hospital Morbidity of the SUS by Place of Hospitalization” dataset, filtered for Piracicaba (Municipality Code: 353870). Admissions were categorized according to the following ICD-10 classifications, set out in Table S1.

Data were recorded by year and month of care, enabling temporal analysis aligned with the study’s defined periods from 2019 to 2024. Hospitalizations represent cases officially registered in the Unified Health System (SUS) for facilities located in Piracicaba.

#### 2.3.2. Physicochemical and Biological Water Variables

Environmental data were obtained from two key sources:

PCJ Situation Room [17]: This system monitors the Cantareira System and associated reservoirs. Flow data for the Piracicaba River, reported in cubic meters per second (m^3^/s), flow (m), and precipitation (mm), were extracted and converted into monthly averages to align with hospitalization data, set out in Table S1.

SEMAE (Municipal Water and Sewage Service) [2]: Additional data on water quality in the source were obtained from bulletins made available by SEMAE.

INMET [18]: The data obtained from INMET made it possible to assess the climate conditions during this period through the bulletins made available, set out in Table S1.

### 2.4. Statistical Analysis

All statistical analyses were conducted using R software (version 4.2.2) within the RStudio environment (version 2024.12.0 + 467) [21]. Initially, the data were organized into two matrices: **X** (health-related variables) and **Y** (physicochemical and biological water variables). The associations between variables were assessed using Spearman’s rank correlation coefficient, a non-parametric measure suitable for capturing monotonic relationships. The statistical significance of the correlations was evaluated through bootstrap resampling with 10,000 iterations, allowing for the construction of 95% confidence intervals. Correlations whose confidence intervals included zero were considered non-significant.

To explore the global relationship between the two matrices, canonical correlation analysis (CCA) was employed to identify patterns of association between the variable sets [22]. Temporal patterns in disease data were examined using hierarchical cluster analysis, based on the Spearman’s correlation matrix derived from the bootstrap simulations. The clustering was performed using the unweighted pair group method with arithmetic mean (UPGMA), and the cluster validity was assessed via the cophenetic correlation coefficient to ensure that the hierarchical structure accurately reflected the original data. These analyses were conducted using the “vegan” package [23].

Following the correlation analyses, the variables in matrix Y were subjected to principal component analysis (PCA) for dimensionality reduction (k = 16). PCA was performed based on the correlation matrix using the “FactoMineR” package [24]. To determine the optimal number of principal components to retain, parallel analysis was conducted using the “psych” package [25]. The retained principal components (PCs) were subsequently included as covariates in generalized linear mixed models (GLMMs), with model parameters estimated via maximum likelihood.

GLMMs were fitted using the “glmmTMB” package [26], adopting a negative binomial distribution to account for overdispersion observed in the disease count data, which exhibited greater variability than expected under a standard Poisson model [27]. In this context, disease counts were modeled considering the retained PCs as fixed effects and including random effects for year and season nested within year to capture longitudinal dependencies. Alternative nested models, incorporating different combinations of covariates in the linear predictor, were compared using likelihood ratio tests.

The general form of the model used to analyze disease counts is represented by the following equation:

(1) $$\log(\mu_{ijk})=\beta_{0}+\sum_{p=1}^{k}{\beta_{p}PC}_{p}+b_{i}+b_{j(i)}$$

where ${µ}_{ijk}$ is the expected mean of disease counts for the k-th observation in the j-th season within the i-th year; $\beta_{0}$ is the intercept; $\beta_{p}$ represents the p-th principal component used as a covariate; $b_{i}∼N(0,\sigma_{year}^{2})$ is the random effect for the i-th year; and $b_{j(i)}∼N(0,\sigma_{season}^{2})$ is the random effect for the j-th season nested within the i-th year. The logarithmic link function (log) ensures that the predicted mean (${µ}_{ijk}$) is positive and appropriate for count data. The model’s variance is adjusted using a dispersion parameter specific to the negative binomial distribution to address overdispersion.

## 3. Results

This section will present data obtained from correlation analysis and longitudinal modeling of the incidence of diseases associated with the physicochemical and biological variables of the water in the Piracicaba River. The results are organized in such a way as to highlight the main relationships between environmental variables (such as pH, temperature, humidity, and the concentration of cyanobacteria, among others) and the occurrence of respiratory, digestive system, nervous system, and other diseases. Firstly, the data obtained will be presented in the form of a minimum, maximum, mean, standard deviation (SD), and range for each variable, as shown in Table 1 and the Supplementary Material Excel document.

In our study, we did not observe any variation in the incidence of experimental diseases or changes in physicochemical evaluations during the period that included the COVID-19 pandemic.

### 3.1. Correlation Analysis Between a Disease and Physicochemical and Biological Water Variables

**Figure 1:**
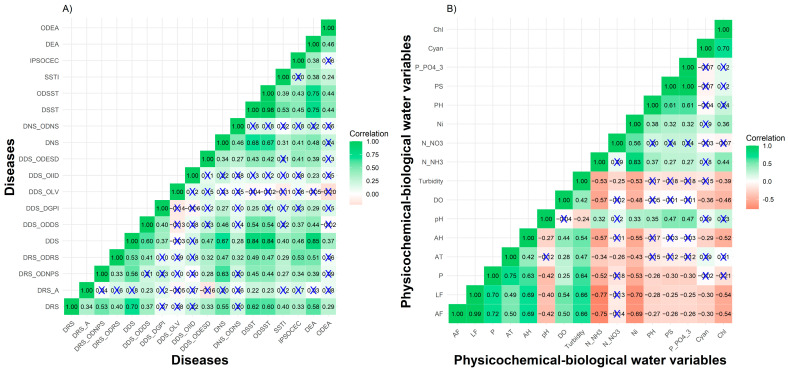
(**A**) A correlation matrix between diseases. (**B**) A correlation matrix between physicochemical and biological water variables. Correlations were calculated using the bootstrap method with 10,000 simulations. Correlations marked with “X” indicate non-significant results. The color gradient reflects the strength and direction of correlations, ranging from −0.5 (red) to 1.0 (green).

The interpretation of the data presented in Figure 1 provides us with support for a deeper understanding of the interactions between human health and the environmental conditions of the Piracicaba River, contributing to the formulation of effective prevention and mitigation strategies. In general, a positive correlation is observed between the diseases, except in the case of DDS_DGPI and DDS_OLV (Figure 1A), which are not related to the others.

In Figure 1B, the correlations between the physicochemical and biological variables show positive and negative patterns; for example, cyanobacteria (Cyan) are negatively correlated with the other variables while the concentration of nitrate (N_NO3) in the water has a negative relationship with turbidity and average temperature (AT). The correlation values presented in Figure 1A,B, along with their respective confidence intervals generated through bootstrap simulation, can be found in Tables S2 and S3.

The canonical correlation analysis identified two significant relationships between the diseases and the physicochemical and biological characteristics of the Piracicaba River (Table 2). The first canonical pair (correlation = 0.90; *p* = 0.00037; Figure S1) indicates that high concentrations of phosphate (P_PO4_3) and lower concentrations of soluble phosphorus (PS) are strongly associated with an increase in the incidence of diseases such as SDB and ODSST. The second canonical pair (correlation = 0.83; *p* = 0.036; Figure S1) reveals that high levels of inorganic nitrogen (Ni) and higher pH are correlated with an increased incidence of diseases such as DSST and ODSST, while lower concentrations of chlorophyll (Chl) indicate waters with lower biological productivity.

**Figure 2:**
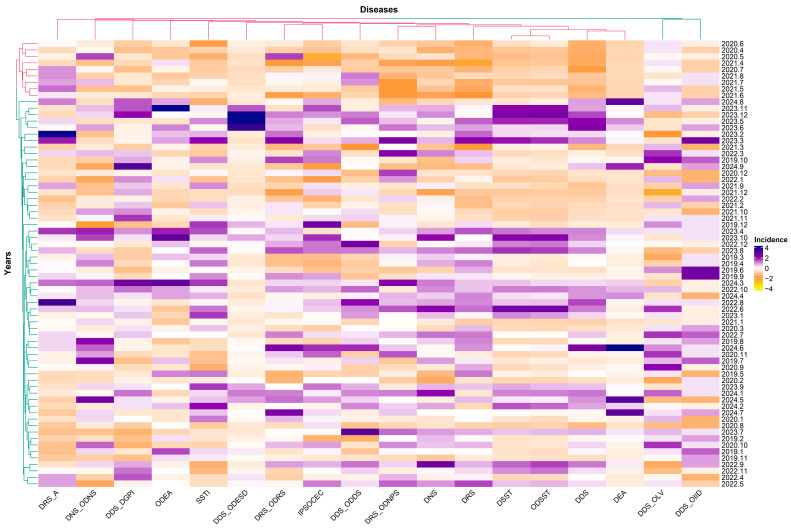
The heatmap showing the temporal variation of disease incidence across different years. The rows represent the years, while the columns represent the diseases. Hierarchical clustering of diseases (**top**) and years (**left**) was performed based on the Spearman’s correlation matrix obtained through 10,000 bootstrap simulations. The UPGMA (unweighted pair group method with arithmetic mean) method was used as the linkage criterion. The color gradient represents the standardized incidence values, ranging from −4 (yellow) to 4 (purple).

Overall, the results indicate a strong relationship between the water quality of the Piracicaba River and the occurrence of diseases, highlighting the role of pollution by nutrients, heavy metals, and physicochemical variations in the spread of health problems. This information is essential for directing environmental policies and monitoring strategies, aiming at improving water quality and reducing impacts on public health. Therefore, the relationship between the incidence of diseases over the years was evaluated to verify the repetition patterns, based on a heatmap. In Figure 2, the colors indicate the incidence levels, with darker shades of purple representing high incidence and shades of orange indicating low incidence.

The colors represent standardized incidence values: yellow indicates values below the average, purple above the average, and white close to the average. The dendrograms at the top and left show the clustering of diseases and years, respectively, based on the similarity of their incidence patterns, calculated by Spearman’s correlation and grouped by the UPGMA method, with cophenetic coefficients of 0.81 (diseases) and 0.91 (years) indicating robust clusters. These clusters highlight diseases with similar behaviors over time and periods with similar incidence patterns.

The heatmap analysis reveals distinct incidence patterns over time. Two well-defined clusters are formed for diseases and two clusters for years, indicating similar epidemiological behaviors within each group. Some diseases show incidence peaks concentrated in specific periods, suggesting possible outbreaks or seasonal variations. In addition, certain years or time intervals cluster together, which may reflect the influence of environmental factors, social factors, or external events that impacted the incidence of multiple diseases simultaneously (Figure 2).

It is worth noting that the diseases DDS_OLV and DDS_OIID, as well as some months of 2020, present atypical incidence patterns in relation to the other diseases and periods analyzed, which suggests specific epidemiological dynamics for these cases (Figure 2). These patterns provide relevant insights for understanding the temporal dynamics of diseases, assisting in the planning of public health surveillance and control actions, focusing on periods and diseases of greatest risk.

### 3.2. Principal Component Analysis of the Physicochemical and Biological Variables of the Piracicaba River

**Figure 3:**
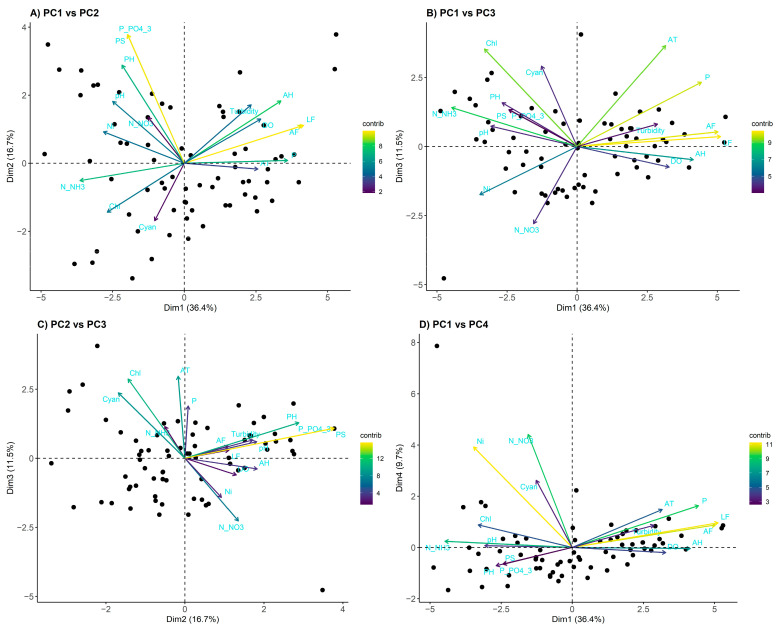
Principal component analysis (PCA) biplots. The graphs represent the projections on the planes: (**A**) PC1 vs. PC2, (**B**) PC1 vs. PC3, (**C**) PC2 vs. PC3, and (**D**) PC1 vs. PC4. The arrows represent the original variables, with direction and length indicating the correlation with the principal components. The colors of the arrows reflect the relative contribution of each variable to the formation of the respective principal axes, following a viridis color scale (from purple to yellow, with higher values indicating greater importance). The analyzed variables include water parameters (pH, dissolved oxygen, turbidity, N-NH₃, N-NO₃, total inorganic nitrogen, total phosphorus, soluble phosphorus, P-PO₄^3−^, cyanobacteria concentration, and chlorophyll), river parameters (flow rate, discharge, and precipitation), and climate parameters (temperature and humidity).

To assess the influence of the physicochemical and biological variables of the Piracicaba River, a principal component analysis (PCA) was performed. The variables considered included water parameters (pH, dissolved oxygen, turbidity, ammonia, nitrate, total inorganic nitrogen, total phosphorus, soluble phosphorus, phosphate, cyanobacterial concentration, and chlorophyll), river parameters (discharge, flow, and precipitation), and climate parameters (temperature and humidity). The selection of principal components was based on parallel analysis (Figure S2), resulting in the retention of the first four components, which together explained 74.3% of the total data variability (Figure 3).

The first principal component (PC1), responsible for 36.4% of the variance, showed correlation with variables related to the river (mean level, mean flow, and precipitation), water parameters (ammonia and inorganic nitrogen), and climate parameters (humidity). The second principal component (PC2), which explained 16.7% of the variability, was associated with water variables, notably phosphate, soluble phosphorus, and pH. The third principal component (PC3), with 11.5% of the variance, was related to river variables (precipitation), water variables (nitrate, inorganic nitrogen, cyanobacteria concentration, and chlorophyll), and climate variables (temperature). Finally, the fourth principal component (PC4), which represented 9.7% of the total variability, was correlated with water variables, specifically nitrate, chlorophyll, and cyanobacteria concentration (Figure S3). These retained PCs were used in the GLMMs to infer the relationships between environmental variables and disease incidence in the city of Piracicaba (Section 3.3, Section 3.4, and Section 3.5).

### 3.3. Diseases of the Respiratory System

Respiratory diseases (DRS, DRS_A, DRS_ODNPS, and DRS_ODRS) presented distinct patterns of influence of environmental and temporal factors on the disease incidence in the city of Piracicaba. The results obtained through GLMM, adjusted with negative binomial distribution, are presented in Table 3. It highlights both the fixed effects, represented by physicochemical and biological variables of the water synthesized in principal components (PCs), and the random effects, attributed to the variability associated with the season and the year alone.

DRS, which encompasses all respiratory diseases, did not show a significant influence of the environmental parameters analyzed. However, a significant influence was observed from the random effects of year/season and year alone, suggesting the presence of relevant temporal variations. For DRS_A, there was a statistically significant association with PC2 (estimate = −0.1378; *p* = 0.0031), indicating that the increase in phosphate, soluble phosphorus, and pH levels is related to a reduction in the incidence of the disease.

The incidence of DRS_ODNPS was significantly influenced by PC1 (estimate = −0.0765; *p* = 0.0032), which aggregates variables related to the river’s hydrological regime (such as mean level, mean flow, and precipitation), water quality (nitrate and inorganic nitrogen), and climatic conditions (relative humidity). These results suggest that higher values of these parameters are associated with a decrease in the occurrence of the disease. On the other hand, for DRS_ODRS, no statistically significant associations were detected with the environmental or temporal factors analyzed, which indicates that its incidence may be related to other determinants not covered in this study.

**Figure 4:**
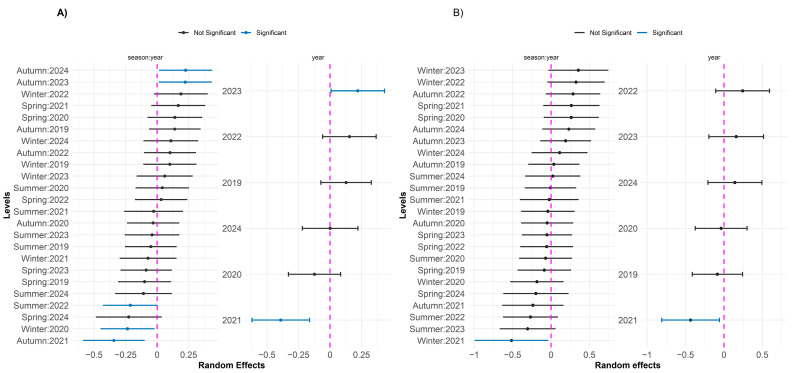
Random effects of GLMMs with a negative binomial distribution for respiratory diseases: (**A**) DRS and (**B**) DRS_ODNPS. The left panels show random effects for the year/season, and the right panels show random effects for the year. Dots represent point estimates, and horizontal bars indicate 90% confidence intervals. Significant effects (blue) have confidence intervals that do not overlap the zero reference line (dashed magenta), while non-significant effects are shown in black.

The significant random effects observed for the diseases DRS and DRS_ODNPS are presented in Figure 4. For DRS, the nested random effect of the season within year was more evident for autumn, indicating a relevant impact on the incidence of the disease, possibly associated with seasonal environmental conditions. In addition, the year 2023 showed a significant positive effect, suggesting an increase in the incidence of DRS, possibly related to extreme environmental events. For DRS_ODNPS, the seasonal variation was less pronounced, with most of the effects not being significant, except for winter 2021 (Winter: 2021), which showed a significant negative effect. The year 2021 also showed a significant negative effect. The other years and seasons showed effects close to zero, suggesting stability in the incidence of DRS_ODNPS over time, with less interannual and seasonal variation compared to DRS.

### 3.4. Diseases of the Digestive System

Digestive system diseases (DDS, DDS_DGPI, and DDS_ODESD) are influenced by the season within the year and by the year in isolation, as shown in Table 4. On the other hand, diseases such as DDS_ODDS, DDS_OLV and DDS_OIID are not related to seasonal changes. In general, when considering all diseases related to the digestive system together (DDS), no influence of the parameters analyzed in this research is observed. However, when analyzing each digestive system disease separately, it is possible to identify correlations with the variables studied.

**Figure 5:**
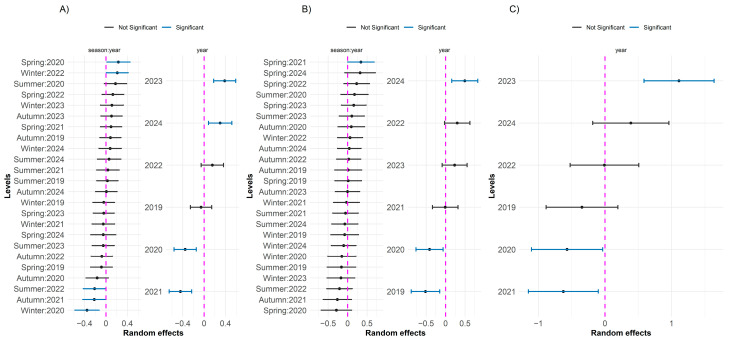
Random effects of GLMMs with a negative binomial distribution for diseases of the digestive system: (**A**) DDS_ODDS, (**B**) DDS_DGPI, and (**C**) DDS_ODESD. Dots represent point estimates, and horizontal bars indicate 90% confidence intervals. Significant effects (blue) have confidence intervals not overlapping the zero reference line (dashed magenta), while non-significant effects are shown in black.

The influence of the physicochemical and biological variables of the Piracicaba River was analyzed in relation to the DDS_ODDS, DDS_DGPI, and DDS_ODESD diseases. For the disease DDS_ODDS, PC2 (estimate = −0.0723; *p* = 0.0002) suggests that the increase in the concentrations of phosphate, soluble phosphorus, and pH is associated with a reduction in the incidence of the disease. On the other hand, for DDS_ODESD, the same PC2 (estimate = 0.2099; *p* = 0.0022) indicated that the increase in these parameters is related to an increase in the incidence of the disease. In addition, PC3 (estimate = 0.1349; *p* = 0.0089) showed that factors such as precipitation, nitrate, inorganic nitrogen, concentration of cyanobacteria, chlorophyll, and temperature are associated with an increase in the incidence of DDS_ODESD. Figure 5 demonstrates the influence of different seasons within the same year and years separately on the average number of cases of DDS, DDS_DGPI, and DDS_ODESD. Different seasons of the year have a greater influence on DDS, specifically from 2020 to 2022. The year 2023 stands out in the scenario of digestive system diseases, suggesting a higher incidence of these diseases, potentially associated with favorable environmental conditions, such as rising temperatures or changes in the water quality of the Piracicaba River. On the other hand, years such as 2020 and 2021 showed significant negative effects, indicating a lower incidence of diseases, which may be associated with events of less environmental stress or greater stability in the parameters of the physicochemical and biological variables of the Piracicaba River.

### 3.5. Diseases of the Nervous System and Other Diseases

In general, diseases of the nervous system and other diseases are associated with the physicochemical and biological variables of the Piracicaba River and also present seasonal patterns, as shown in Table 5. However, some diseases, such as DNS, DNS_ODNS, DSST, ODSST, and ODEA, are not influenced by the fixed factors represented by the PCs. In addition, the incidence of these diseases varies according to specific years.

The variables PC1 (estimate = −0.0407; *p* = 0.0122) and PC2 (estimate = −0.0590; *p* = 0.0460) showed a negative relationship with the incidence of SSTI, indicating that the increase in the variables associated with these components may be related to the reduction in the occurrence of this disease. On the other hand, PC3 showed a positive relationship with IPSOCEC (estimate = 0.0189; *p* = 0.0305) and DEA (estimate = 0.1192; *p* = 0.0451) diseases, suggesting that the increase in the variables included in this component may be associated with an increase in the occurrence of these diseases. PC4 (estimate = −0.0198; *p* = 0.0361), which includes variables such as nitrate, chlorophyll, and cyanobacteria concentration, showed a negative effect on the incidence of IPSOCEC.

**Figure 6:**
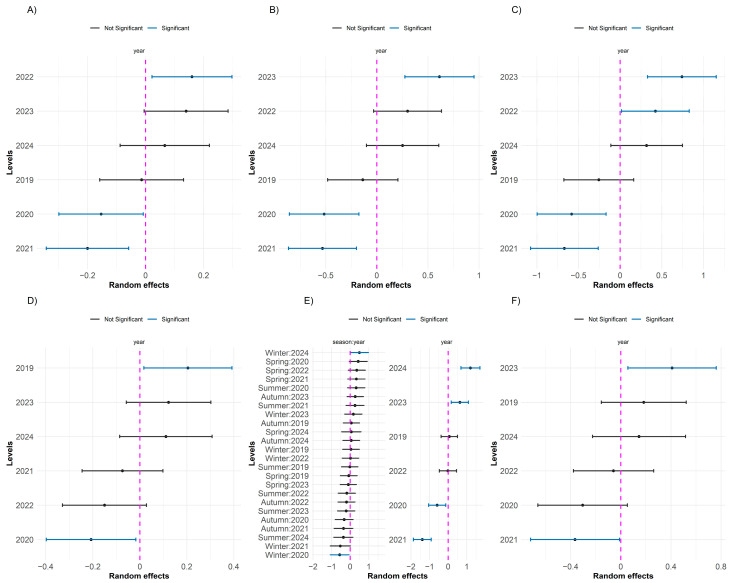
Random effects from GLMMs with a negative binomial distribution for nervous system diseases: (**A**) DNS, and other diseases: (**B**) DSST, (**C**) ODSST, (**D**) SSTI, (**E**) DEA, and (**F**) ODEA. Dots represent point estimates, and horizontal bars indicate 90% confidence intervals. Significant effects (blue) have confidence intervals that do not overlap the zero reference line (dashed magenta), while non-significant effects are shown in black.

The random effects estimated by the GLMM models indicated significant variations in the incidence of diseases over the years (Figure 6). For diseases of the nervous system, such as DNS, a significant negative effect was observed in 2020 and 2021 and a positive effect in 2022, suggesting the influence of environmental factors or changes in exposure conditions between these years.

Similar trends were observed for DSST and ODSST, with significant positive effects in 2023 and 2022, respectively, while ODSST showed a negative effect in 2020. These results point to a possible trend of increasing incidence of these diseases in more recent years, possibly related to environmental changes, such as fluctuations in water quality or climate change.

A similar pattern of temporal variation was observed for other diseases. In the case of DEA, the year 2019 showed a significant positive effect, contrasting with the negative effect observed in 2020. For ODEA, the year 2023 stood out with a significant positive effect, indicating an increase in the occurrence of the disease in that period.

## 4. Discussion

Environmental pollution has worsened over the years, possibly due to improper disposal of industrial waste, agricultural waste, improper discharge of effluents, and emerging compounds, among others. As a result, water bodies are gradually deteriorating, affecting the local population and causing various respiratory and digestive diseases, among others. As demonstrated in the study by Hanif et al. [28], water pollution in the Kapotaksha River impacts the health of the population: for example, 4% are affected by diarrhea, 5% are affected by dysentery, 25% of people suffer from respiratory diseases, and 4% suffer from asthma [28].

We observed variations in the values found for the physicochemical and biological parameters that reflect the deterioration of water quality. Comparing these values with the limits of the National Environmental Council (CONAMA), Resolution 357/2005, and the Ministry of Health, Ordinance GM/MS No. 888/2021, for fresh water intended for human consumption, we noticed that the concentration of cyanobacteria and chlorophyll are above the reference value, and in addition, we observed that dissolved oxygen presents values below the expected (Table S4) [29,30]. In this context, based on statistical analysis, we were able to predict which environmental or water quality patterns may affect the population of Piracicaba (Table 1). These data and results obtained enable planning for the SUS, where changes in this parameter may indicate a greater demand for the health system in the region. Therefore, the cross-referencing and correlation of these data allow us to have a broader view of the influence of the Piracicaba River on the local population, thus being a crucial factor for the city’s annual health planning.

### 4.1. Diseases of the Respiratory System

Respiratory system diseases (DRS and DRS_ODNPS) are influenced by the seasons/year, as shown in Table 3. These diseases are affected by meteorological variables, airborne allergens, and air pollution. Therefore, climatic factors (temperature, humidity, wind speed, and storms, among others) can affect the components of the atmosphere through their interaction with the airways, inducing clinical respiratory symptoms. In addition, contamination by respiratory viruses tends to occur during the colder seasons, especially when low temperatures are accompanied by dry conditions. The disease called DRS_ODRS is not related to the change in season/year because these types of diseases are classified as post-surgical diseases, syndromes, disorders, and respiratory failures, among others, and are thus affected by other external factors [16,31,32].

DRS, in general, when all diseases related to the respiratory system are added together, are not influenced by the parameters analyzed in this research. However, as the diseases of the respiratory system are observed separately, a correlation with the variables studied can be found.

DRS_A is negatively influenced by PC2. When pH conditions are optimal and in accordance with physiological conditions, we observe a decrease in this disease. However, any disturbance causing changes in the pH of the water can irritate the nasal mucous membranes when inhaled [33,34,35]. The concentration of phosphorus in water can increase serum concentrations in humans, resulting in a decrease in the incidence of asthma cases, according to a study by Changhai et al. [36]. The lack of micronutrients, such as phosphorus, for example, is associated with a higher incidence of atopic diseases, according to a review by Peroni et al. [37].

The DRS_ODNPS has a negative influence on PC1. The conditions of the river, together with changes in the climate, can directly influence some lung diseases due to variations in relative humidity, reducing, for example, calcification of the nasal cavities or ulceration. Humidity exerts a primary control on fire occurrence and, therefore, on respiratory disease since particulate matter emissions decrease [33,34,35,38,39]. The presence of nitrogen may aid in lung cell metabolism, acid-base balance, and human lung ecology. Thus, we can observe from the data obtained that this chemical element and its compounds can be incorporated into amides, amino acids, proteins, nucleic acids, vitamins, etc., or as energy producers [40].

The increase in the incidence of DRS in 2023 may be associated with the bloom event that occurred in the region during this period where a degradation in water quality and an increase in the concentration of cyanobacteria and cyanotoxins were observed, which resulted in fish mortality [2,5].

### 4.2. Diseases of the Digestive System

Digestive system diseases (DDS, DDS_DGPI, and DDS_ODESD) are influenced by the seasons/year, as shown in Table 4, due to changes in eating habits, physical activities, behavior, and the immune system, among others [41,42]. In the systematic study by Fares [41], the author observes that seasonal peaks in digestive system diseases, such as peptic ulcers, are more prominent in the colder months, while the peak incidence rate of Crohn’s disease in most countries was found during the spring and summer seasons. The seasonal trend in the onset of acute pancreatitis shows a summer peak in some countries.

On the other hand, the diseases called DDS_ODDS, DDS_OLV, and DDS_OIID were not related to the change in season/year.

DDS in general, when all diseases related to the digestive system are added together, are not influenced by the parameters analyzed in this research. However, as diseases of the digestive system are observed separately, a correlation with the variables studied can be found.

DDS_ODESD has a positive influence on PC2. Excess nutrients, especially phosphorus, cause a greater proliferation of pathogenic organisms that release toxins into the water. As a result, the efficiency of the processes used in water treatment plants (WTP) may be compromised, thus increasing the incidence of diseases related to the digestive system [34,35,43]. Cyanotoxins, for example, are a diverse group of hepato-, neuro-, cyto-, and dermatotoxins, including microcystins and nodularins, which inhibit serine/threonine protein phosphatases and lead to hepatic system, neurological, immunological, and reproductive damage that can be potentially fatal. Their physiological effects vary between organisms and depend on the route of exposure, with effects ranging from skin irritation and nausea to tissue necrosis and death. An example of cyanotoxins that affect the digestive system are anabaenapeptins [40]. In addition, the release of potentially toxic chemical elements into water suffering from hypoxia can be aggravated by increasing the ingestion and inhalation of these chemical elements. pH influences the acidity of the digestive system, which can cause irritation and changes in its functioning [34,35,44]. DDS_ODDS, on the other hand, has a negative influence on PC2. This observed evidence may be correlated with the types of diseases included in this classification, which are diseases related to food intolerance, intestinal malformation, and post-surgical syndromes, among others. Therefore, pH directly influences these diseases by causing physiological changes that improve phosphorus absorption [45]. With the increase of this chemical element in the body, we can observe that there may be a decrease in several diseases, including chronic renal failure, malabsorption of the small intestine, and vitamin D-deficient osteomalacia [Peacock].

DDS_DGPI, on the other hand, has a positive influence on PC3. The presence of nitrates and nitrites in water can trigger gastritis, ulcers, and diarrhea, among others. This factor is influenced by precipitation and temperature, together with nitrogen, which can trigger flowering phenomena, increasing the incidence of the disease [39,44].

The diseases DDS_OLV and DDS_OIID do not show any correlation with the variables studied, and the occurrence of these diseases is correlated with other parameters, which were not the focus of this research. In order to find diseases related to the liver and other intestinal infections, the population of Piracicaba should have had prolonged contact with river water, so no effect of the variables on these diseases was observed.

### 4.3. Diseases of the Nervous System and Other Diseases

Diseases of the nervous system vary with seasonality, as shown in Table 5, due to changes in several environmental factors, with an increase in diseases related to acute stroke, meningitis, encephalitis, Guillain-Barré syndrome, and demyelinating disease [46]. For example, changes in seasons, which vary between the optimal temperature, can aggravate deaths resulting from the nervous system [47]. Skin diseases are also aggravated by rising temperatures, including atopic dermatitis, measles and eczema, infectious skin diseases, and skin cancer [48,49]. The same is observed for other diseases where climatic factors and pollution aggravate the incidence of these diseases [50,51].

SSTI has a negative correlation with PC1 and PC2. The river conditions and relative humidity help maintain healthy skin. Thermal and humid comfort can prevent dry skin, preventing possible related diseases, such as itching, pruritus, and cracks [52,53]. In addition to humidity, the ideal pH maintains the skin microbiota, preventing possible infectious processes or skin degradation [53].

IPSOCEC and DEA have a positive influence on PC3. Excess nutrients can cause algal blooms, which, in contact with toxins present in the water from recreational activities in bodies of water, can result in itching, allergic reactions, irritation of the eyes and mucous membranes, and diarrhea, among others [54,55]. The proliferation of pathogenic organisms that release toxins into water, thus compromising the efficiency of the processes used in water treatment plants (WTPs), increases the incidence of diseases [34,35,43]. Cyanotoxins, for example, are a diverse group of hepato-, neuro-, and cytotoxic chemicals. Their physiological effects vary among organisms and depend on the route of exposure, with effects ranging from nausea, tissue necrosis, and death [44]. Furthermore, the release of potentially toxic chemicals into the water suffering from hypoxia can be aggravated by increasing the ingestion and inhalation of these chemicals. The pH influence on acidity or alkalinity can compromise proper physiological functioning [34,35,43].

### 4.4. Future Directions

Our studies were based on the hypothesis that aerosols formed along the Piracicaba River influence the incidence of diseases in the population of Piracicaba. However, throughout the study and after the elaboration of this research, there is room for studying the effects of river water on the incidence of other diseases related to the aquatic environment, viruses, bacteria, and other organisms. In addition, other factors can be added as variations to scientific studies, such as city expansion, concentration of green areas, and agricultural advances, among others.

## 5. Conclusions

This study highlights the significant impact of environmental factors, particularly variations in river quality and climate, on public health outcomes in urban settings. When analyzing hospital admission rates in Piracicaba over the period from 2019 to 2024, clear seasonal patterns emerged. Changes in parameters such as phosphate and soluble phosphorus concentration, pH, river level and flow, and air humidity influence the incidence of diseases of the respiratory system. Diseases of the digestive system are influenced by the parameters of phosphate and soluble phosphorus concentration, pH, nitrate and inorganic nitrogen concentration, cyanobacteria concentration, chlorophyll, and temperature. Finally, skin and eye diseases show variation in their incidence when there is a variation in phosphate and soluble phosphorus concentration, pH, nitrate and inorganic nitrogen concentration, cyanobacteria concentration, chlorophyll, and temperature. The findings highlight:

(i) Seasonal health risks: High hospital admissions during contamination peaks highlight the vulnerability of populations living near or interacting with polluted water sources.
(ii) Implications for public health policy: Enhanced environmental monitoring and timely public health warnings are essential to mitigate risks during periods of high contamination. Enhanced health system preparedness is critical to effectively address seasonal spikes in hospital admissions.
(iii) Studies that correlate physicochemical and biological parameters are needed to understand disease incidence as well as their effects on humans.

Despite its limitations, this study provides valuable insights into the intersection of environmental and health systems. Future research should aim to include direct exposure measurements, new longitudinal analyses to deepen our understanding of acute and chronic impacts, and evidence-based strategies to protect vulnerable populations and promote sustainable management of urban rivers.

## Figures and Tables

**Table 1:** Descriptive statistics of disease variables and physicochemical and biological water parameters (PBW) monitored in the Piracicaba River basin between 2019 and 2024. The table presents the minimum, maximum, mean, standard deviation (SD), and range for each variable.

Variable	Domain	Min	Max	Mean	SD	Range
DRS	Disease	56	321	192.8406	59.3352	265.0000
DRS_A	Disease	1	37	8.6377	6.5078	36.0000
DRS_ODNPS	Disease	1	51	23.5362	11.0435	50.0000
DRS_ODRS	Disease	15	49	30.1739	7.7724	34.0000
DDS	Disease	103	671	347.8406	131.5954	568.0000
DDS_ODDS	Disease	14	69	35.5362	10.7095	55.0000
DDS_DGPI	Disease	1	20	6.9130	4.0791	19.0000
DDS_OLV	Disease	2	14	8.3478	2.6392	12.0000
DDS_OIID	Disease	1	8	3.5362	1.7199	7.0000
DDS_ODESD	Disease	1	86	11.8696	15.6627	85.0000
DNS	Disease	26	89	51.6522	13.9493	63.0000
DNS_ODNS	Disease	2	21	10.5942	3.9494	19.0000
DSST	Disease	18	203	77.3768	47.1163	185.0000
ODSST	Disease	12	181	62.4638	44.6258	169.0000
SSTI	Disease	4	27	14.8841	5.8650	23.0000
IPSOCEC	Disease	214	373	289.3913	31.1388	159.0000
DEA	Disease	2	745	110.9420	135.3495	743.0000
ODEA	Disease	1	22	4.8986	3.7735	21.0000
AF	PBW	15	216	66.6022	48.3878	200.1700
LF	PBW	1	3	1.5646	0.4919	2.0500
P	PBW	13	248	98.3890	61.2965	234.7800
AT	PBW	17	27	222,630	2.6199	9.9000
AH	PBW	34	83	65.7413	10.9020	48.8500
pH	PBW	7	8	7.3117	0.2076	1.1000
DO	PBW	1	5	2.9617	0.9753	4.5500
Turbidity	PBW	3	478	36.1996	80.5959	474.7800
N_NH3	PBW	0	14	3.5042	2.4983	13.1300
N_NO3	PBW	0	39	3.2591	4.5323	38.5000
Ni	PBW	2	43	6.7635	5.2471	40.2800
PH	PBW	0	2	0.7926	0.4127	1.7600
PS	PBW	0	1	0.4418	0.2720	1.0620
P_PO4_3	PBW	0	3	1.3529	0.8340	3.2500
Cyan	PBW	1943	122,480	17,167.2899	18,618.2456	120,537.0000
Chl	PBW	7	145	39.3371	33.4134	138.3800

**Table 2:** Standardized coefficients of canonical pairs between disease variables (X) and physicochemical and biological water variables (PBW) of the Piracicaba River (Y).

Diseases (X)	PBW (Y)	First Canonical Pair		Second Canonical Pair	
		X Can1	Y Can1	X Can2	Y Can2
DRS	AF	1.1154	−1.459	−0.1674	−0.9173
DRS_A	LF	−0.2186	1.0894	0.5188	0.7965
DRS_ODNPS	P	−0.4309	0.3952	−0.0246	−0.3262
DRS_ODRS	AT	0.1153	−0.5485	0.3079	−0.0159
DDS	AH	−0.1943	−0.1272	−0.1064	−0.1788
DDS_ODDS	pH	0.0188	−0.1819	0.4909	−0.2355
DDS_DGPI	DO	−0.0162	−0.2238	−0.389	−0.0324
DDS_OLV	Turbidity	0.0828	−0.1021	−0.0423	−0.1115
DDS_OIID	N_NH3	0.0766	0.1881	−0.2058	−1.2125
DDS_ODESD	N_NO3	0.2984	0.7135	−0.0307	−0.3094
DNS	Ni	−0.0304	−0.7545	−0.0619	0.826
DNS_ODNS	PH	−0.1402	0.1609	−0.1306	0.7079
DSST	PS	−0.7364	−3.2199	1.691	0.7986
ODSST	P_PO4_3	0.8604	2.5739	−1.1225	−0.8186
SSTI	Cyan	0.0391	−0.0752	0.0142	0.1643
IPSOCEC	Chl	−0.1094	0.0529	−0.5083	−0.7244
DEA	-	−1.2629	-	0.1506	-
ODEA	-	−0.0418	-	0.0639	-
Correlation		0.90		0.83	
*p*-value		0.00037		0.036	

**Table 3:** Results of the generalized linear mixed models (GLMM) with negative binomial distribution for respiratory diseases.

Diseases	Significant Fixed Effects	Year/Season (Random Effect)	Year (Random Effect)
DRS	-	✓	✓
DRS_A	PC2 ↓	-	-
DRS_ODNPS	PC1 ↓	✓	✓
DRS_ODRS	-	-	-

✓ has an effect.

**Table 4:** Results of the generalized linear mixed models (GLMM) with negative binomial distribution digestive system.

Diseases	Significant Fixed Effects	Year/Season (Random Effect)	Year (Random Effect)
DDS	-	✓	✓
DDS_ODDS	PC2 ↓	-	-
DDS_DGPI	PC3 ↑	✓	✓
DDS_OLV	-	-	-
DDS_OIID	-	-	-
DDS_ODESD	PC2 ↑	-	✓

✓ has an effect.

**Table 5:** Results of the generalized linear mixed models (GLMM) with negative binomial distribution for nervous system and other diseases.

Diseases	Significant Fixed Effects	Year/Season (Random Effect)	Year (Random Effect)
Diseases nervous system			
DNS	-	-	✓
DNS_ODNS	-	-	-
Other diseases			
DSST	-	-	✓
ODSST	-	-	✓
SSTI	PC1↓ and PC2 ↓	-	✓
IPSOCEC	PC3 ↑ and PC4 ↓	-	-
DEA	PC3 ↑	✓	✓
ODEA	-	-	✓

✓ has an effect.

## Data Availability

Data will be made available upon request (Excel sheets with data and official documents).

## Supplementary Materials

The following supporting information can be downloaded at: https://www.mdpi.com/article/10.3390/toxics13050359/s1, Figure S1: Canonical scores between the sets of disease-related variables (X) and physicochemical and biological water parameters (Y) derived from canonical correlation analysis (CCA). (A) First canonical pair (Can 1), showing a strong correlation between the canonical variates (r = 0.90, *p* = 0.00037). (B) Second canonical pair (Can 2), also presenting a strong canonical correlation (r = 0.83, *p* = 0.036). Linear regression lines (in magenta) illustrate the association between the canonical scores. Figure S2: Parallel analysis of the principal component analysis (PCA) of the physicochemical and biological variables. Continuous line corresponds to PC actual data (blue), and dashed line (red) corresponds to PC resampled data; Figure S3: Contributions of variables (%) to (A) PC1, (B) PC2, (C) PC3, and (D) PC4. The analyzed variables include water parameters (pH, dissolved oxygen, turbidity, N-NH₃, N-NO₃, total inorganic nitrogen, total phosphorus, soluble phosphorus, P-PO_4_^3−^, cyanobacteria concentration, and chlorophyll), river parameters (flow rate, discharge, and precipitation), and climate parameters (temperature and humidity). Table S1: Acronyms of the diseases and parameters studied. Table S2. Spearman’s correlation matrix for diseases. Table S3: Spearman’s correlation matrix for physicochemical and biological water variables. Table S4: Freshwater quality standards according to CONAMA and the Brazilian Ministry of Health.

## Abbreviations

The following abbreviations are used in this manuscript:

DRS	Diseases of the Respiratory System
DRS_A	Diseases of the Respiratory System_Asthma
DRS_ODNPS	Diseases of the Respiratory System- Other Diseases of the Nose and Paranasal Sinuses
DRS_ODRS	Diseases Of The Respiratory System_ Other Diseases Of The Respiratory System
DDS	Diseases of the Digestive System
DDS_ODDS	Diseases of the Digestive System_ Other Diseases of the Digestive System
DDS_DGPI	Diseases of the Digestive System_ Diarrhea and Gastroenteritis of Presumed Infectious Origin
DDS_OLV	Diseases of the Digestive System_ Other Liver Diseases
DDS_OIID	Diseases of the Digestive System_ Other Infectious Intestinal Diseases
DDS_ODESD	Diseases of the Digestive System_ Other Diseases of the Esophagus, Stomach and Duodenum
DNS	Diseases of the Nervous System
DNS_ODNS	Diseases of the Nervous System_ Other Diseases of the Nervous System
DSST	Diseases of the Skin And Subcutaneous Tissue
ODSST	Other Diseases of the Skin And Subcutaneous Tissue
SSTI	Skin and Subcutaneous Tissue Infections
IPSOCEC	Injuries, Poisoning and Some Other Consequences of External Causes
DEA	Diseases of the Eye and Adnexa
ODEA	Other Diseases of the Eye and Adnexa
AF	Average Flow (m^3^/s)
LF	Average Level Flow (m)
P	Average Precipitation (mm)
AT	Average Temperature (°C)
W	Wind, Average Speed (m/s)
AH	Average Relative Air Humidity (%)
pH	pH
DO	Dissolved Oxygen (mg/L O_2_)
NTU	Turbidity (UNT)
NH	N_NH_3 (mg/L)
NO	N_NO_3 (mg/L)
N	Ntotal Inorganic (mg/L)
PH	Ptotal (mg/L)
PS	Psoluble (mg/L)
PO	_PO4_3 (mg/L)
Cyn	Cyanobacteria (n° cel/mL)
Chl	Chlorophyll (mg/L)
CETESB	Environmental Company of the State of São Paulo
SEMAE	Municipal Water and Sewage Services
BOD	Biochemical Oxygen Demand
TDS	Total Dissolved Solids
LPS	Lipopolysaccharides
SUS	Unified Health System
DATASUS	Unified Health System’s Information Technology Department
INMET	National Institute of Meteorology
PCJ	Comitês das Bacias Hidrográficas dos Rios Piracicaba, Capivari e Jundiaí e Comitê da Bacia Hidrográfica dos Rios Piracicaba e Jaguari.
ICD-10	International Classification of Diseases
CCA	canonical correlation analysis
UPGMA	Unweighted Pair Group Method with Arithmetic Mean
PCA	Principal Component Analysis
PC	Principal Components
