# Supplementary material for: Correlation Analysis Between Physical–Chemical and Biological Conditions in the River and the Incidence of Diseases in the City of Piracicaba, Brazil
Source: Toxics. 2025 Apr 30;13(5):359. doi: 10.3390/toxics13050359 (PMC12115644; doi:10.3390/toxics13050359)
Supplement: Supplementary file 1 [file toxics-13-00359-s001.zip › Suplementary_Material_Toxics_.pdf]

SUPLEMENTARY MATERIAL

Correlation analysis between physicalchemical and biological conditions in the river and the incidence of diseases in the city of Piracicaba, Brazil

Alexander Ossanes de Souza[1], Deoclecio Jardim Amorim[1] and Ernani Pinto[1,*]

[1]Center of Nuclear Energy in Agriculture, Av. Centenário, 303 - São Dimas, 13416-000, Piracicaba-SP, University of São Paulo, Brazil

*Corresponding author: e-mail: ernani@usp.br, phone: + 55 11 30911505

Table S1: Acronym of the diseases and parameters studied

Diseases	Acronym	Parameters	Acronym
Diseases of the Respiratory System	DRS	Average Flow (m3/s)	AF
Diseases of the Respiratory System - Asthma	DRS_A	Average Level Flow (m)	LF
Diseases of the Respiratory System - Other Diseases of the Nose and Paranasal Sinuses	DRS_ODNPS	Average Precipitation (mm)	P
Diseases Of The Respiratory System - Other Diseases Of The Respiratory System	DRS - ODRS	Average Temperature (°C)	AT
Diseases of the Digestive System	DDS	Wind, Average Speed (m/s)	W
Diseases of the Digestive System - Other Diseases of the Digestive System	DDS_ODDS	Average Relative Air Humidity (%)	AH
Diseases of the Digestive System - Diarrhea and Gastroenteritis of Presumed Infectious Origin	DDS_DGPI	pH	pH
Diseases of the Digestive System - Other Liver Diseases	DDS_OLV	Dissolved Oxygen (mg/L O2)	DO
Diseases of the Digestive System - Other Infectious Intestinal Diseases	DDS_OIID	Turbidity (UNT)	NTU
Diseases of the Digestive System - Other Diseases of the Esophagus, Stomach and Duodenum	DDS_ODESD	N-NH3 (mg/L)	NH
Diseases of the Nervous System	DNS	N-NO3 (mg/L)	NO
Diseases of the Nervous System - Other Diseases of the Nervous System	DNS_ODNS	Ntotal Inorganic (mg/L)	N

Diseases of the Skin And Subcutaneous Tissue	DSST	Ptotal (mg/L)	PH
Other Diseases of the Skin And Subcutaneous Tissue	ODSST	Psoluble (mg/L)	PS
Skin and Subcutaneous Tissue Infections	SSTI	P-PO4-3 (mg/L)	PO
Injuries, Poisoning and Some Other Consequences of External Causes	IPSOCEC	Cyanobacteria (n° cel/mL)	Cyn
Diseases of the Eye and Adnexa	DEA	Chlorophyll (mg/L)	Chl
Other Diseases of the Eye and Adnexa	ODEA		

Table S2. Spearman correlation matrix among disease variables, accompanied by their respective 95% confidence intervals derived from bootstrap resampling (10,000 iterations).

	AF	LF	P	AT	AH	pH	DO	Turbidity	N_NH3	N_NO3	Ni	PH	PS	P_PO4_3	Cyan	Chl
**AF**	1	0.99 [0.97, 1]	0.72 [0.61, 0.82]	0.5 [0.29, 0.68]	0.69 [0.53, 0.81]	-0.42 [-0.59, -0.22]	0.5 [0.28, 0.69]	0.66 [0.51, 0.79]	-0.75 [-0.85, -0.63]	-0.14 [-0.37, 0.09]	-0.69 [-0.79, -0.56]	-0.27 [-0.5, -0.01]	-0.26 [-0.46, -0.03]	-0.26 [-0.46, -0.03]	-0.3 [-0.53, -0.04]	-0.54 [-0.7, -0.34]
**LF**		1	0.7 [0.57, 0.8]	0.49 [0.28, 0.67]	0.69 [0.53, 0.81]	-0.4 [-0.58, -0.19]	0.54 [0.33, 0.71]	0.66 [0.51, 0.79]	-0.77 [-0.86, -0.65]	-0.13 [-0.36, 0.12]	-0.7 [-0.8, -0.57]	-0.28 [-0.51, -0.02]	-0.25 [-0.46, -0.02]	-0.25 [-0.46, -0.02]	-0.3 [-0.52, -0.05]	-0.54 [-0.7, -0.35]
**P**			1	0.75 [0.63, 0.84]	0.63 [0.48, 0.75]	-0.42 [-0.63, -0.18]	0.25 [0.01, 0.48]	0.64 [0.48, 0.76]	-0.52 [-0.67, -0.34]	-0.18 [-0.43, 0.09]	-0.53 [-0.68, -0.37]	-0.26 [-0.47, -0.04]	-0.3 [-0.51, -0.06]	-0.3 [-0.51, -0.06]	-0.02 [-0.26, 0.22]	-0.21 [-0.41, 0.02]
**AT**				1	0.42 [0.2, 0.62]	-0.12 [-0.35, 0.12]	0.28 [0.07, 0.47]	0.47 [0.28, 0.65]	-0.34 [-0.53, -0.12]	-0.26 [-0.48, -0.03]	-0.43 [-0.63, -0.21]	-0.15 [-0.37, 0.07]	-0.12 [-0.34, 0.11]	-0.12 [-0.34, 0.11]	0.09 [-0.14, 0.31]	0.01 [-0.2, 0.24]
**AH**					1	-0.27 [-0.48, -0.04]	0.44 [0.23, 0.62]	0.54 [0.36, 0.69]	-0.57 [-0.73, -0.38]	-0.11 [-0.34, 0.12]	-0.55 [-0.7, -0.37]	-0.17 [-0.39, 0.06]	-0.03 [-0.29, 0.23]	-0.03 [-0.29, 0.23]	-0.29 [-0.52, -0.04]	-0.52 [-0.69, -0.31]
**pH**						1	-0.04 [-0.27, 0.21]	-0.24 [-0.46, -0.01]	0.32 [0.09, 0.53]	0.12 [-0.17, 0.4]	0.33 [0.1, 0.54]	0.35 [0.12, 0.55]	0.47 [0.24, 0.67]	0.47 [0.24, 0.67]	0.09 [-0.17, 0.33]	0.23 [-0.02, 0.45]
**DO**							1	0.42 [0.18, 0.62]	-0.57 [-0.72, -0.38]	-0.02 [-0.26, 0.23]	-0.48 [-0.63, -0.3]	-0.05 [-0.29, 0.2]	-0.01 [-0.27, 0.24]	-0.01 [-0.27, 0.24]	-0.36 [-0.57, -0.13]	-0.46 [-0.66, -0.25]
**Turbidity**								1	-0.53 [-0.7, -0.33]	-0.25 [-0.47, -0.02]	-0.53 [-0.7, -0.34]	-0.17 [-0.4, 0.09]	-0.18 [-0.42, 0.06]	-0.18 [-0.42, 0.06]	-0.15 [-0.38, 0.09]	-0.39 [-0.57, -0.18]
**N_NH3**									1	0.09 [-0.16, 0.33]	0.83 [0.74, 0.89]	0.37 [0.13, 0.59]	0.27 [0.02, 0.51]	0.27 [0.02, 0.51]	0.18 [-0.08, 0.43]	0.44 [0.2, 0.64]
**N_NO3**										1	0.56 [0.36, 0.73]	0.2 [-0.04, 0.43]	0.24 [-0.03, 0.51]	0.24 [-0.03, 0.51]	-0.03 [-0.27, 0.21]	-0.07 [-0.31, 0.18]
**Ni**											1	0.38 [0.16, 0.59]	0.32 [0.08, 0.55]	0.32 [0.08, 0.55]	0.19 [-0.07, 0.44]	0.36 [0.13, 0.57]
**PH**												1	0.61 [0.42, 0.78]	0.61 [0.42, 0.78]	-0.04 [-0.26, 0.19]	0.24 [-0.01, 0.47]
**PS**													1	1 [1, 1]	-0.07 [-0.29, 0.16]	0.12 [-0.14, 0.38]
**P_PO4_3**														1	-0.07 [-0.29, 0.16]	0.12 [-0.14, 0.38]
**Cyan**															1	0.7 [0.56, 0.81]
**Chl**																1

Table S3: Correlation matrix Spearman between physicochemical and biological water variables, accompanied by their respective 95% confidence intervals derived from bootstrap resampling (10,000 iterations).

	DRS	DRS_A	DRS_ODNPS	DRS_ODRS	DDS	DDS_ODDS	DDS_DGPI	DDS_OLV	DDS_OIID	DDS_ODESD	DNS	DNS_ODNS	DSST	ODSST	SSTI	IPSOCEC	DEA	ODEA
**DRS**	1	0.34 [0.09, 0.57]	0.53 [0.34, 0.7]	0.4 [0.17, 0.6]	0.7 [0.53, 0.84]	0.37 [0.15, 0.56]	0.07 [-0.16, 0.29]	0.08 [-0.15, 0.3]	0.22 [-0.01, 0.45]	0.33 [0.09, 0.54]	0.55 [0.35, 0.72]	0.2 [-0.04, 0.44]	0.62 [0.45, 0.77]	0.6 [0.42, 0.75]	0.4 [0.18, 0.59]	0.33 [0.1, 0.54]	0.58 [0.36, 0.76]	0.29 [0.05, 0.51]
**DRS_A**		1	0.04 [-0.22, 0.29]	0.16 [-0.07, 0.38]	0.18 [-0.06, 0.41]	0.23 [0, 0.45]	0.12 [-0.14, 0.36]	-0.05 [-0.28, 0.18]	0.07 [-0.19, 0.33]	-0.16 [-0.38, 0.08]	0.2 [-0.02, 0.41]	0.06 [-0.2, 0.31]	0.22 [0, 0.43]	0.23 [0.02, 0.44]	0.12 [-0.11, 0.34]	0.17 [-0.05, 0.37]	0.03 [-0.21, 0.27]	0.08 [-0.17, 0.32]
**DRS_ODNPS**			1	0.33 [0.12, 0.52]	0.56 [0.37, 0.72]	0.21 [-0.03, 0.43]	0.23 [-0.02, 0.46]	0.1 [-0.15, 0.34]	0 [-0.24, 0.25]	0.28 [0.03, 0.51]	0.63 [0.46, 0.77]	0.2 [-0.05, 0.44]	0.45 [0.23, 0.64]	0.44 [0.23, 0.62]	0.27 [0.02, 0.49]	0.34 [0.13, 0.54]	0.39 [0.15, 0.6]	0.09 [-0.16, 0.33]
**DRS_ODRS**				1	0.53 [0.33, 0.69]	0.41 [0.2, 0.6]	0.1 [-0.13, 0.32]	0.09 [-0.18, 0.35]	0.18 [-0.08, 0.42]	0.32 [0.09, 0.53]	0.47 [0.26, 0.66]	0.32 [0.09, 0.53]	0.49 [0.29, 0.66]	0.47 [0.27, 0.65]	0.29 [0.06, 0.5]	0.53 [0.32, 0.71]	0.51 [0.31, 0.68]	0.06 [-0.17, 0.28]
**DDS**					1	0.6 [0.42, 0.75]	0.37 [0.17, 0.56]	-0.03 [-0.26, 0.2]	0.18 [-0.06, 0.39]	0.47 [0.25, 0.66]	0.67 [0.52, 0.8]	0.28 [0.05, 0.49]	0.84 [0.78, 0.9]	0.84 [0.77, 0.9]	0.4 [0.19, 0.59]	0.46 [0.27, 0.64]	0.85 [0.76, 0.92]	0.37 [0.14, 0.58]
**DDS_ODDS**						1	0.4 [0.17, 0.6]	-0.13 [-0.36, 0.11]	0.08 [-0.16, 0.31]	0.23 [-0.01, 0.45]	0.46 [0.27, 0.62]	0.14 [-0.1, 0.36]	0.54 [0.34, 0.7]	0.54 [0.34, 0.71]	0.22 [-0.02, 0.44]	0.37 [0.14, 0.58]	0.44 [0.24, 0.62]	-0.02 [-0.25, 0.21]
**DDS_DGPI**							1	-0.14 [-0.37, 0.11]	-0.06 [-0.29, 0.18]	0.12 [-0.12, 0.35]	0.27 [0.03, 0.5]	0.1 [-0.15, 0.35]	0.25 [0.02, 0.46]	0.21 [-0.02, 0.43]	0.17 [-0.07, 0.4]	0.23 [-0.01, 0.47]	0.29 [0.06, 0.49]	0.15 [-0.09, 0.37]
**DDS_OLV**								1	0.12 [-0.11, 0.34]	0.15 [-0.09, 0.37]	0.03 [-0.23, 0.28]	0.05 [-0.2, 0.29]	-0.04 [-0.28, 0.2]	-0.02 [-0.26, 0.22]	-0.11 [-0.35, 0.12]	0.06 [-0.19, 0.3]	-0.05 [-0.26, 0.16]	-0.2 [-0.4, 0.01]
**DDS_OIID**									1	0.11 [-0.13, 0.33]	0.22 [-0.01, 0.43]	0.08 [-0.16, 0.31]	0.13 [-0.1, 0.34]	0.12 [-0.12, 0.34]	0.1 [-0.13, 0.33]	0.08 [-0.15, 0.31]	0.23 [0.01, 0.43]	0.05 [-0.16, 0.26]
**DDS_ODESD**										1	0.34 [0.12, 0.53]	0.27 [0.01, 0.5]	0.43 [0.2, 0.63]	0.42 [0.18, 0.62]	0.21 [-0.01, 0.42]	0.41 [0.2, 0.59]	0.39 [0.14, 0.6]	0.13 [-0.12, 0.37]
**DNS**											1	0.46 [0.26, 0.64]	0.68 [0.53, 0.79]	0.67 [0.53, 0.78]	0.31 [0.08, 0.52]	0.41 [0.2, 0.6]	0.48 [0.27, 0.66]	0.24 [-0.02, 0.47]
**DNS_ODNS**												1	0.16 [-0.07, 0.39]	0.18 [-0.05, 0.4]	0.02 [-0.22, 0.27]	0.18 [-0.09, 0.44]	0.22 [-0.03, 0.45]	0.06 [-0.2, 0.3]
**DSST**													1	0.98 [0.95, 0.99]	0.53 [0.35, 0.69]	0.45 [0.24, 0.62]	0.75 [0.64, 0.84]	0.44 [0.2, 0.64]
**ODSST**														1	0.39 [0.17, 0.58]	0.43 [0.23, 0.61]	0.75 [0.64, 0.84]	0.44 [0.2, 0.64]
**SSTI**															1	0.2 [-0.04, 0.42]	0.38 [0.16, 0.58]	0.24 [0, 0.46]
**IPSOCEC**																1	0.38 [0.16, 0.57]	0.06 [-0.19, 0.3]
**DEA**																	1	0.46 [0.26, 0.64]
**ODEA**																		1

Table S4: Freshwater quality standards according to CONAMA and the Brazilian Ministry of Health

	CONAMA Resolution n° 357/2005			Ministry of Health Ordinance GM/MS nº 888
	Class 1	Class 2	Class 3	Surface Water Supply Sources
pH	6 a 9	6 a 9	6 a 9	6 a 9
DO	> 6 mg/L O_2	> 5 mg/L O_2	> 4 mg/L O_2	-
NUT	40 NUT	100 NUT	100 NUT	-
$N\text{-}NH_3$	3.7mg/L to pH £ 7.5<br>2.0 mg/L to 7.5 < pH £ 8.0<br>1.0 mg/L to 8.0 < pH £ 8.5<br>0.5 mg/L to pH > 8.5	3.7mg/L to pH £ 7.5<br>2.0 mg/L to 7.5 < pH £ 8,0<br>1.0 mg/L to 8.0 < pH £ 8.5<br>0.5 mg/L to pH > 8,5	13.3 mg/L to pH £ 7.5<br>5.6 mg/L to 7.5 < pH £ 8.0<br>2.2 mg/L to 8.0 < pH £ 8.5<br>1.0 mg/L to pH > 8.5	-
$N\text{-}NO_3$	10.0 mg/L	10.0 mg/L	10 mg/L	10 mg/L
PH	0.1 mg/L	0.1 mg/L	0.15 mg/L	-
Cyn	20.000 cel/mL	50000 cel/mL	100000 cel/mL	£ 10.000 cel/mL
Chl	10 μg/L	30 μg/L	60 μg/L	10 μg/L

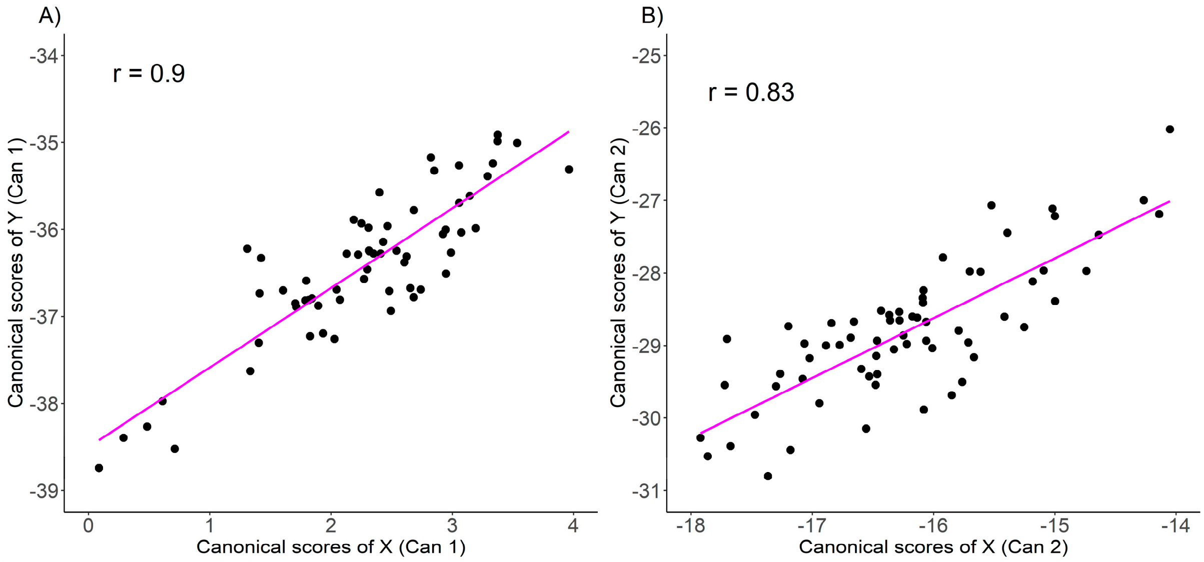

Figure S1 Canonical scores between the sets of disease-related variables (X) and physicochemical and biological water parameters (Y) derived from canonical correlation analysis (CCA). **(A)** First canonical pair (Can 1), showing a strong correlation between the canonical variates (r = 0.90, p = 0.00037). **(B)** Second canonical pair (Can 2), also presenting a strong canonical correlation (r = 0.83, p = 0.036). Linear regression lines (in magenta) illustrate the association between the canonical scores.

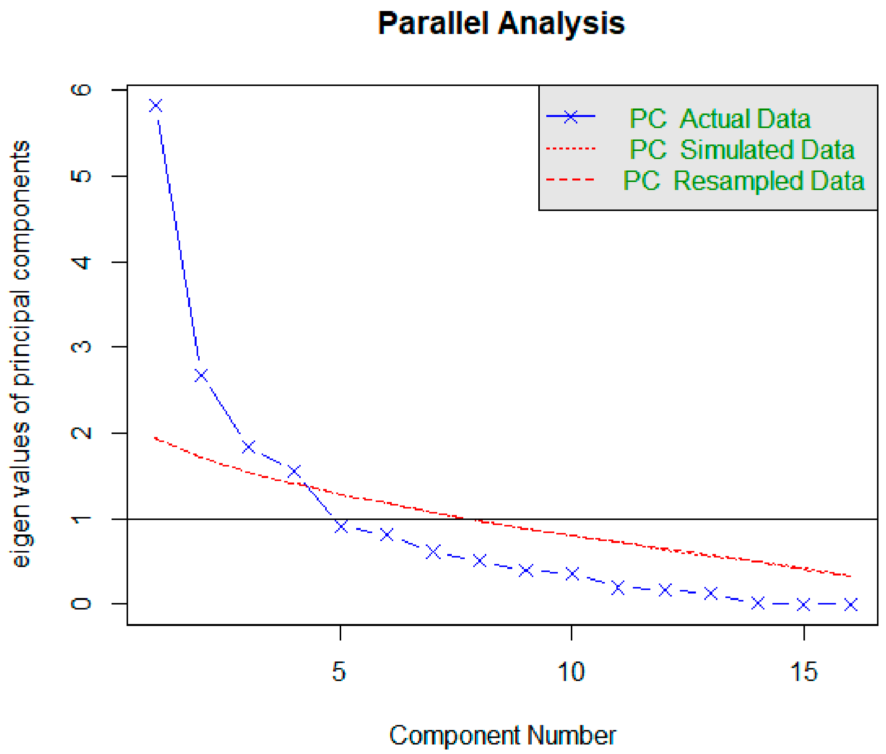

Figure S2 Parallel Analysis of the Principal Components Analysis (PCA) of the physical-chemical-biological variables. Continuous line corresponds to PC actual data (blue), and dashed line (red) corresponds to PC resampled data.

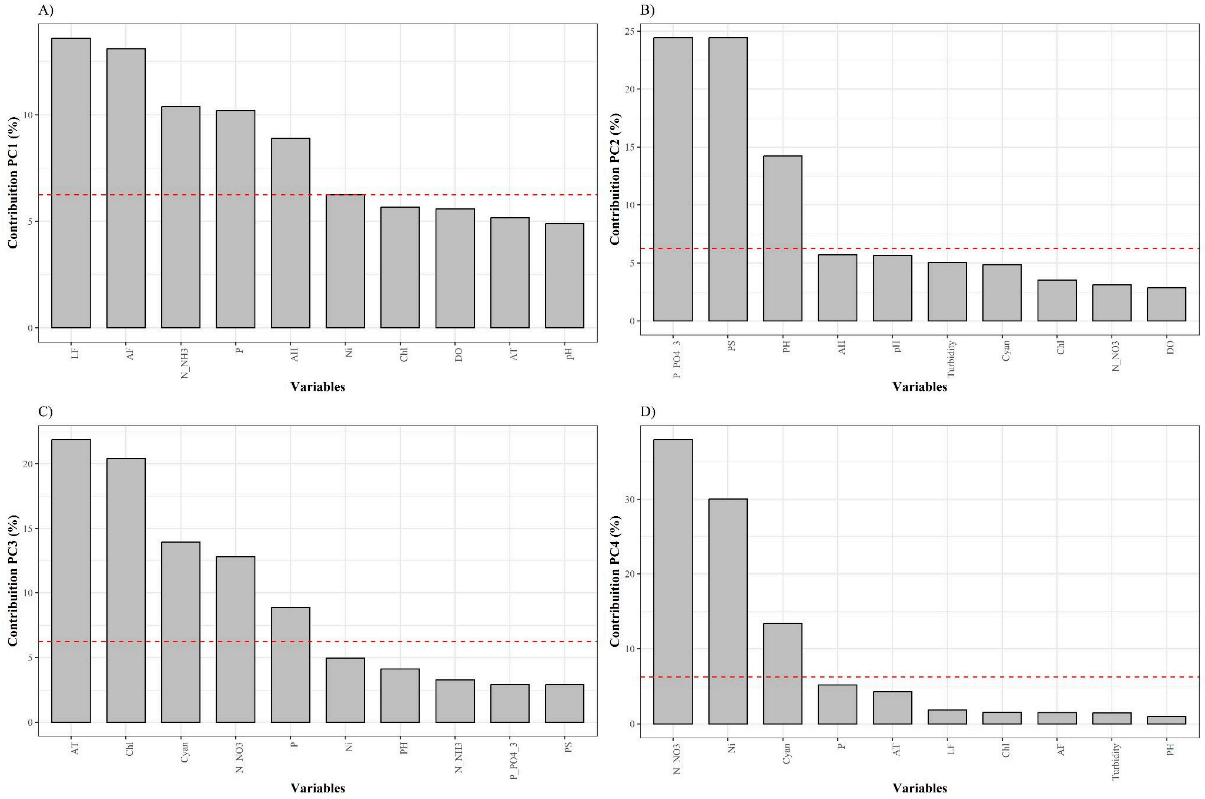

Figure S3 Contributions of variables (%) to **A)** PC1, **B)** PC2, **C)** PC3, and **D)** PC4. The analyzed variables include water parameters (pH, dissolved oxygen, turbidity, N-NH_3, N-NO_3, total inorganic nitrogen, total phosphorus, soluble phosphorus, P-PO_4^{3-}, cyanobacteria concentration, and chlorophyll), river parameters (flow rate, discharge, and precipitation), and climate parameters (temperature and humidity).
